# Relationship between employment values and college students’ choice intention of slow employment: A moderated mediation model

**DOI:** 10.3389/fpsyg.2022.940556

**Published:** 2022-08-11

**Authors:** Ting Wang, Shiqing Li

**Affiliations:** School of Business, China University of Political Science and Law, Beijing, China

**Keywords:** college students’ employment, choice intention of slow employment, employment values, employment anxiety, social support

## Abstract

This study examines the impact of employment values on college students’ choice intention of slow employment, as well as the role of employment anxiety and social support in this process. A questionnaire survey is conducted among students from several universities in Haidian and Changping Districts of Beijing to test a theoretical model. It is found that the employment long-term income orientation and employment cost avoidance orientation significantly positively predicted the choice intention of slow employment, and employment anxiety plays a mediating role in this relationship. The short-term income orientation of employment significantly negatively affects the choice intention of slow employment. Social support moderates the impact of the long-term and short-term employment income orientation on employment anxiety and the mediation effect of employment anxiety. This study contributes to the literature on college students’ employment psychology and behavior, and it provides an approach for colleges and universities, society at large, families, and college students to reduce the choice intention of slow employment and achieve fuller employment.

## Introduction

Employment among college students is related to the high-quality development of the social economy and higher education. The employment psychology and behavior of college students have changed with changes in the socioeconomic environment. Recently, the phenomenon of slow employment has received attention. Slow employment means that, after graduation, college students neither make efforts to gain employment nor plan to study further, but instead make other choices, such as staying at home, studying abroad, caring for their parents, preparing for various vocational exams, or following some other non-employment direction (Ma and Deng, 2019; Zhang, 2021; Xiu and Xu, 2022). The slow employment of college students in China is not the rest between high school education and university education, but the wandering between the highest degree education and the first employment, which is mainly reflected in the delay of the first employment time and the delay of the overall employment process (Song et al., 2021). As the number of college graduates grows every year, their employment prospects are becoming increasingly poor, and they are also coming under increasing employment pressure. To alleviate these issues, many college students choose slow employment or even non-employment (Tang and Sun, 2021). According to the 2022 employment analysis report on college students released by Zhaopin Ltd.,^1^ 15.9% of college students choose slow employment. The number of students opting for slow employment is increasing in historical terms. The slow employment of college students gradually evolves from an individual phenomenon to an increasingly common group phenomenon. Continuous structural contradictions and uncertainties, such as those owing to the COVID-19 pandemic, have exacerbated this phenomenon, making it more difficult for college students to achieve fuller and higher-quality employment (Zeng, 2020). The experience of slow employment over the long term will accustom several college graduates to a state of unemployment, reduce their employment enthusiasm, and increase the possibility of developing so-called lazy employment or delaying employment for a long time (Wang, 2020). In order to cope with the severe situation of college students’ employment, the state has put stabilizing employment in a more prominent position and issued various assistance policies. Colleges, universities, and the society have also actively created conditions for graduates’ employment. However, the spreading phenomenon of slow employment has hindered the process of overall employment, and will also aggravate the contradiction between supply and demand in the labor market, result in a waste of talents, affect the social outlook on talents, and have a certain adverse impact on the colleges, families, and individuals (Wang, 2021; Xu, 2021). Whether from the perspective of individuals or countries, means of reducing college students’ choice intention of slow employment to alleviate the group phenomenon of slow employment has become a research problem that is worthy of attention.

Living conditions and employment values have changed drastically among contemporary college students, and this is the main reason for slow employment (Zeng, 2021). Employment values refer to the views and behavioral tendencies relating to job characteristics or attributes in relation to employment (Pan, 2014; Xu, 2019). Realistically, in China, the uncertain employment environment and collectivist values promote the extensive formation of employment values to support career stability and roll into the system after COVID-19 (Liu and Zhang, 2021), which largely affects the time and space of college students’ employment choices and behaviors (Jiang, 2020). The changes in these employment choices demonstrate an increasing trend in college students’ risk aversion (Li, 2020). Establishing a proper concept for job choice and employment values can be an effective way of alleviating slow employment. How do employment values of college students affect their willingness to choose slow employment and through what mechanism? How can college students adjust their value orientations and adapt themselves to the new employment environment? How should colleges, families, and society at large help college students control the spread of slow employment? Currently, there has been no systematic and clear analysis or study of these problems. Using the self-verification theory, this study explores the impact of employment values on the choice intention of slow employment. Drawing on the extant literature and the feelings-as-information theory, this study takes employment anxiety as a mediating variable to investigate the mediating mechanism between employment values and the choice intention of slow employment. In addition, this study also explores the moderating role of social support.

There are three theoretical implications of this study. First, this study explores the influence mechanism between employment values and the choice intention of slow employment from the perspective of college students’ behavior psychology, which complements the specific research on slow employment from the perspective of individual psychology. Second, this study divides employment values into long-term employment income orientation, short-term employment income orientation, and employment cost avoidance orientation, which expandes the dimensions of employment values, and made the dimension research of employment values more specific. Finally, the moderating role of social support shows that the application of social support theory varies with different characteristic groups, which makes a certain exploration for the application scope of social support theory. The practical implication of this study is to inspire college students to reduce their willingness to choose slow employment by adjusting their employment values and Employment anxiety, so as to achieve more full employment. It also inspires families, colleges and society to provide appropriate support for college students to alleviate the phenomenon of slow employment.

## Literature review and research hypothesis

College students’ employment values are closely associated with employment intention and graduation destination, an area that is attracting increasing attention in the employment-related research. The employment values of college students have changed with time, exhibiting the distinct characteristics of different times (Wang, 2021). Born between 1970 and 1980, college students tended to emphasize traditional factors, such as job stability, salary, and welfare (Guo and Liu, 2005). Born in the 1990s, college students focused more on fashion factors, such as personal interests, and less on traditional factors, such as income and welfare. They usually preferred nearby cities and attached greater importance to value stability and became less willing to take risks (Chang, 2013). Born after 1995, college students’ job-hunting preparation improved, they became more self-evident regarding their job-hunting intentions, and their job-hunting objectives were diversified. However, several problems also arose in the employment values of college students who graduated after 1995: their self-awareness became too strong, they exhibited a contradictory employment mentality, and their dedication and service consciousness weakened (Xiao and Zhang, 2019). Affected by the uncertainty in the environment owing to the epidemic and other issues, college students now tend to make more conservative employment decisions or choose slow employment to avoid risks. Their employment values are certainly rolled into the system, reflected by the increased importance of job stability and tendency of the system toward unit selection (Liu and Zhang, 2021). Employment values have a significant impact on college students’ employment quality (Zhong and Liu, 2015), employment anxiety (Wang et al., 2014), initial employment results (Yin and Fan, 2015), job satisfaction and career development (Yarbrough et al., 2016), career decision-making self-efficacy (Doo and Park, 2019; Kamaruddin and Rasdi, 2021), and so on. Currently, numerous employment values of college students are characterized by subjectivity and realism, and their employment expectations are high, resulting in reduced employment satisfaction. Therefore, to guide the establishment of scientific values, we should strengthen ideal vocational and employment guidance education (Tan and Feng, 2017). Achieving the goal of college students’ employment values education requires the joint efforts of multiple educational agents, such as colleges and universities, families, and the government; furthermore, it requires different synergies to promote the integration of employment values education and overall education system (Qu, 2015). Previous studies have indicated the importance of employment values for college students’ employment and highlighted the academic value of employment values in the field of employment research (Pang, 2016). For today’s employment problems, such as high pressure on the total employment of college students, mismatch between the demand and supply structure, and the impact of the external environment, an in-depth study of employment values also has important practical significance.

The existing research divides the slow employment into positive slow employment and negative slow employment. Positive slow employment emphasizes initiative, which means that graduates have a clear understanding and goal of themselves, and use a period of time after graduation to learn skills, improve skills, and prepare for future employment; Negative slow employment refers to the temporary unemployment of graduates due to slow career planning, insufficient skills and no desire for employment (Xu, 2021; Xiu and Xu, 2022). Chinese scholars’ study of the slow employment of college students primarily emphasizes the causes and countermeasures. College students are in the phase of career exploration. They have both longing and hesitation for employment. The phenomenon of slow employment comes into being in this combination. The main reasons for this are the imbalance between the demand and supply for talent in the market, change in college students’ ideal cognition of career, and insufficient effective supply of employment services (Jiang and Liu, 2020). Overall, the causes of slow employment phenomenon are multidimensional, involving not only the individual and family levels but also the university and social levels (Peng et al., 2021). Owing to the COVID-19 pandemic, employment demand fell. Expanding the total employment demand to achieve a balance between demand and supply can help alleviate slow employment (Liu, 2019). The conflict between traditional and modern employment values is an important reason for slow employment on the individual level (Gao, 2021). Therefore, to ameliorate slow employment, it is essential to effectively change the employment concept of college graduates and enable them to actively adapt to the new employment format (Ma and Deng, 2019). Different guidance should be given to college students experiencing positive slow employment versus those experiencing negative slow employment, effective guidance and services should be provided in combination with the actual situation, and various forces of individuals, families, schools, and society should be combined to improve the employment quality of college students (Zheng and Pan, 2019).

The study of slow employment mainly analyzes the current situation and causes of slow employment in an overall perspective, and it highlights corresponding countermeasures. Numerous studies have been conducted on the impact of employment values on employment in research fields related to college students’ employment. However, in relation to the development of the times, college students’ employment values present diversified characteristics and demonstrate new characteristics of the times. Slow employment has become an option for college students after graduation, in addition to employment and enrollment, and college students’ own employment values have become an important influencing factor of slow employment (Zhang, 2020). Hofstede (2011) segregated a dimension of cultural values into long- and short-term orientations. A long-term orientation culture focuses on the future, and a short-term orientation culture tends to consider the past and present more. Pan (2014) divided employment values into employment income and cost. Following Pan (2014), this study introduces long-term and short-term orientations, and divides employment values into long-term employment income, short-term employment income, and employment cost avoidance orientations. The self-verification theory holds that to maintain self-consistency, individuals prefer information consistent with their self-concept (Booth et al., 2020). To realize long-term benefits, college students who prefer long-term employment income may improve themselves in the short term or spend more time planning their career, and may choose slow employment. College students who prefer short-term employment income are more likely to obtain employment directly to realize their short-term income quickly. If college students are willing to expend time and money and pay other costs for employment, they will have a higher willingness to find employment and a lower willingness to choose slow employment. Therefore, this study proposes the following hypotheses:

H1: There is a significant positive relationship between Employment value orientation and choice intention of slow employment.

H1a: There is a significant positive relationship between Long-term employment income orientation and choice intention of slow employment.

H1b: There is a significant negative relationship between Short-term employment income orientation and choice intention of slow employment.

H1c: There is a significant positive relationship between Employment cost avoidance orientation and choice intention of slow employment.

Employment anxiety refers to an unpleasant and painful emotional state, including tension and impatience (Chen et al., 2020), owing to college students’ inability to meet employment goals or expectations when facing job selection and employment challenges. Moreover, it is a state of anxiety (Vignoli, 2015). In a highly competitive employment environment, college students’ employment values are a major cause of their employment anxiety (Ning, 2012). With the lower level of experience of social practices among college students, some maintain an idealistic attitude when they graduate. The gap between reality and ideal will aggravate psychological conflict and produce employment anxiety, while college students with clearer short-term goals and those who are aware of reality will respond to each other more actively, resulting in a lower level of employment anxiety (Liang and Liang, 2012). Feelings-as-information theory believes that people will take their own emotional feelings as the source of information, and then affect individual judgment, cognitive style, and decision-making (Schwarz and Clore, 1983). Therefore, we believe that college students’ Employment anxiety will have an impact on their choice intention of slow employment. College students who are more anxious about employment are less able to fully prepare for employment under high-level employment anxiety. To reduce anxiety, they reduce the frequency of their attempts at career exploration and are more likely to choose slow employment (Vignoli et al., 2005). On the contrary, the more fully prepared, the clearer the goal, and the more confident the employment, the less anxious the college students are, and the less likely they are to choose slow employment. Therefore, this study proposes the following hypotheses:

H2: Employment anxiety is the mediator of employment values and choice intention of slow employment.

H2a: Employment anxiety is the mediator of long-term employment income orientation and choice intention of slow employment.

H2b: Employment anxiety is the mediator of short-term employment income orientation and choice intention of slow employment.

H2c: Employment anxiety is the mediator of employment cost avoidance orientation and choice intention of slow employment.

Social support refers to the support provided by parents, teachers, relatives, friends, and classmates, including material and spiritual support (Chen et al., 2018). The main effect model of social support holds that no matter what level of social support an individual is currently in, as long as social support is increased, the individual’s mental health will be improved (Cohen and Wills, 1985). The buffer model of social support holds that social support has a protective effect on individuals, and usually acts as a buffer for negative emotions (Klineberg et al., 2006). Especially under external stress conditions, social support can buffer the negative effects of stress events on individual physical and mental health. The social support theory of anxiety holds that when individuals encounter difficulties and setbacks, an effective social support system can improve individual mental health and reduce the experience of anxiety, depression and other bad emotions (Porter and Chambless, 2017). Social support in real life is significantly related to reducing depression, anxiety, and social isolation (Meshi and Ellithorpe, 2021). There is a negative correlation between social support in employment and employment anxiety (Chen et al., 2020). When college students face employment problems, the lack of employment support is a main reason behind anxiety (Zhang and Chen, 2006). Actively seeking help and support from parents, teachers, and friends is an effective way of reducing employment pressure and alleviating the negative emotions owing to employment pressure (Wu and Lu, 2013). The more social support college students receive, the greater is the calm with which they face the employment problem to find a solution, and the more easily they can prevent the generation of employment anxiety. From the perspective of theory and literature, the relationship between employment values and Employment anxiety may be different with different levels of social support, and the mediation effect of Employment anxiety may also change. Therefore, this study proposes the following hypotheses:

H3: Social support moderates the relationship between employment values and employment anxiety.

H3a: Social support moderates the relationship between long-term employment income orientation and employment anxiety.

H3b: Social support moderates the relationship between short-term employment income orientation and employment anxiety.

H3c: Social support moderates the relationship between employment cost avoidance orientation and employment anxiety.

H4: Social support moderates the mediation effects of employment anxiety in the relationship between employment values and choice intention of slow employment.

H4a: Social support moderates the mediation effects of employment anxiety in the relationship between long-term employment income orientation and choice intention of slow employment.

H4b: Social support moderates the mediation effects of employment anxiety in the relationship between short-term employment income orientation and choice intention of slow employment.

H4c: Social support moderates the mediation effects of employment anxiety in the relationship between employment cost avoidance orientation and choice intention of slow employment.

**FIGURE 1:**
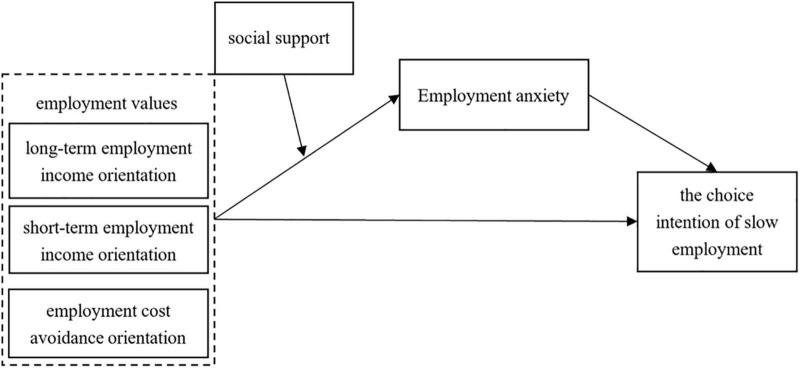
Theoretical research model.

To summarize, this study constructs a moderated mediation model to present the relationship between employment values, employment anxiety, social support, and the choice intention of slow employment. The theoretical model is illustrated in Figure 1.

## Materials and methods

### Research samples

College students studying in the Haidian and Changping Districts of Beijing were investigated using a questionnaire survey. The participants were provided the option of a paper questionnaire or an online questionnaire. A total of 747 questionnaires were distributed, and 638 valid responses were received, for an effective recovery rate of 85.41%. The respondents comprised 298 boys with 340 girls and 103 freshmen, 255 sophomores, 185 juniors, and 95 seniors. In terms of school classification, there were 61 people in Project 985 universities, 179 people in Project 211 universities (excluding Project 985 universities), 137 people in key universities (excluding Projects 985 and 211 universities), 169 people in ordinary universities and 92 people in other institutions. By major, 138 students focused on science and engineering, 277 on economics and management, 144 on the humanities, and 79 on other areas.

### Variable measurement

Employment values are divided into three employment value orientations: long-term employment income orientation, short-term employment income orientation, and employment cost avoidance orientation. Adopting the measurements of employment values developed by Pan (2014), the measurement of long-term employment income orientation includes work–life balance, work stability, development space, professional counterparts, and interests; the measure of short-term income orientation of employment includes starting salary and welfare, working environment and place, nature of unit, and settlement of household registration; and the employment cost avoidance orientation is measured by the reverse willingness of willing to pay employment cost, including avoidance of employment time and money. A 7-point scale is used to measure the importance of each item in employment for subjects, and the mean value reflects the tendency for each orientation. In this study, the coefficient of Cronbach’s α is 0.821.

Using the definition of slow employment, eight items are designed: the overall choice intention of slow employment; the willingness to have free time; study tours; participation in training; support for education; preparation for entrepreneurship; preparation for exams; and spending time, energy, and money for slow employment. Responses are obtained on a 7-point scale, and its mean value reflects the intensity of the choice intention of slow employment. The greater the value is, the greater the willingness to choose slow employment is. In this study, the coefficient of Cronbach’s α is 0.899.

The employment anxiety scale of Zhang and Yao (2011) is used to measure employment anxiety. There are 24 items in total. Each item describes a state of employment anxiety, and the degree of compliance is selected according to their actual situation. A 5-point scale is used here. The average value reflects the degree of employment anxiety of college students. The greater the value is, the greater the degree of anxiety is. In this study, the coefficient for Cronbach’s α is 0.974.

The measurement of social support refers to the scale modified by Li and Wu (2010) from the Xiao Shuiyuan social support scale, adopting a total of 10 items. The subjects respond to the items to describe their current situation, An example question is “How many close friends do you have who can get support and help?” According to the scoring rules implemented by Xiao Shuiyuan, the total score reflects the total level of social support. The larger the total score is, the greater the level of social support is. In this study, the coefficient of Cronbach’s α is 0.678.

### Analytical procedure

First, the reliability and validity of the data were tested. Mplus7 was mainly used here. Second, IBM SPSS Statistics 26.0 was used to analysis the correlations among study variables, the relationship between employment values and the choice intention of slow employment, the mediating effect of employment anxiety about employment values between the choice intention of slow employment, and the moderating role of social support in the relationship between employment values and employment anxiety and in the mediating effect of employment anxiety.

### Discriminant validity of variables

To test the degree of deviation of the common method, the Harman single factor test was used. The results of unrotated factor analysis indicate that the variation interpretation amount of the first factor is 29.899%, which is less than 40%. Thus, there is no factor that can explain most variation, and the danger posed by common method bias is not serious. Furthermore, using confirmatory factor analysis of these data, the discriminant validity of the variables can be identified through model comparison. Table 1 elucidates that the modified six factor model has the best fitting effect, indicating that each factor has good discriminant validity, and the representativeness of the six factors is also good.

**TABLE 1:** Confirmative factor analysis (CFA) results.

Model	x^2^	d*f*	x^2^/d*f*	CFI	TLI	RMSEA	SRMR
Six-factor	3414.864	1519	2.248	0.913	0.908	0.044	0.053
Five-factor	4491.765	1529	2.938	0.864	0.858	0.055	0.093
Four-factor	4813.329	1533	3.140	0.849	0.843	0.058	0.102
Three-factor	5333.926	1536	3.473	0.825	0.818	0.062	0.082
Two-factor	8083.990	1538	5.256	0.699	0.687	0.082	0.101
Single-factor	9988.728	1539	6.490	0.611	0.597	0.093	0.111

## Results

### Descriptive statistics

The descriptive statistical results are presented in Table 2. The long-term employment income orientation and employment cost avoidance orientation are significantly positively correlated with employment anxiety and slow employment choice intention. The short-term employment income orientation exhibits no significant negative correlation with employment anxiety, and it has a significant negative correlation with the choice intention of slow employment. There is a significant positive correlation between employment anxiety and the choice intention of slow employment. All the correlation results obtained here are consistent with the proposed hypotheses.

**TABLE 2:** Descriptive statistics and correlations between all variables (*N* = 638).

Variable	M ± SD	1	2	3	4	5	6	7	8	9	10
1. Sex	1.53 ± 0.50	1									
2. Grade	2.43 ± 0.93	0.031	1								
3. School	2.92 ± 1.23	0.076*	0.047	1							
4. Major	2.26 ± 0.93	–0.011	–0.112***	–0.091**	1						
5. Long-term employment income orientation	5.24 ± 0.93	–0.030	–0.041	0.069*	–0.046	1					
6. Short-term employment income orientation	5.07 ± 1.11	0.035	0.025	0.147***	–0.141***	0.507***	1				
7. Employment cost avoidance orientation	2.69 ± 1.03	–0.074*	–0.007	–0.256***	0.146***	0.001	0.220***	1			
8. Employment anxiety	3.07 ± 0.90	–0.054	–0.034	–0.068*	0.133***	0.093**	–0.053	0.211***	1		
9. Social support	35.2 ± 5.74	–0.004	–0.006	0.067*	–0.063	0.015	0.031	0.143***	–0.506***	1	
10. Choice intention of slow employment	4.54 ± 1.40	–0.126***	–0.088**	–0.266***	0.192***	0.277***	–0.275***	0.472***	0.358***	–0.120***	1

*p < 0.1, **p < 0.05, ***p < 0.01.

### Hypothesis verification

First, Model 4, provided in the process macro program, was used to control gender, grade, school type, and major category to test the direct and mediation effects. The results are reported in Table 3. The long-term employment income orientation significantly positively predictes the choice intention of slow employment (β = 0.30, *P* < 0.01) and employment anxiety (β = 0.10, *P* < 0.05), and employment anxiety significantly positively predictes the choice intention of slow employment (β = 0.29, *P* < 0.01). The bootstrap method indicates that employment anxiety playes a partial mediating role between employment long-term income orientation and choice intention of slow employment, ab = 0.03, SE = 0.01 (90% confidence interval [0.01, 0.05]), and the mediation effect accounts for 10% of the total effect. The short-term employment income orientation significantly negatively predicts the choice intention of slow employment (β = –0.22, *P* < 0.01); however, it does not significantly predict employment anxiety (β = –0.03, *P* > 0.1). Employment anxiety demonstrates no significant mediation effect between the short-term employment income orientation and choice intention of slow employment (90% confidence interval [–0.03, 0.01]). The employment cost avoidance orientation significantly predictes the choice intention of slow employment (β = 0.46, *P* < 0.01) and employment anxiety (β = 0.13, *P* < 0.01); employment anxiety significantly positively predicts the choice intention of slow employment (β = 0.27, *P* < 0.01). The bootstrap method, indicated that employment anxiety plays a partial mediating role between the employment cost avoidance orientation and slow employment choice intention, ab = 0.04, SE = 0.01 (90% confidence interval [0.02, 0.06]), and the mediation effect accounts for 8.70% of the total effect. H1a, H1b, H1c, H2a, and H2c are thus verified.

**TABLE 3:** Mediating effect test results.

	Choice intention of slow employment		Employment anxiety		Choice intention of slow employment	
	β	*t*	β	*t*	β	*t*
Sex	–0.09	−2.62***	–0.04	–1.13	–0.08	−2.38**
Grade	–0.04	–1.14	–0.01	–0.29	–0.04	–1.11
School	–0.26	−7.28***	–0.06	–1.51	–0.24	−7.15***
Major	0.18	4.89***	0.13	3.30***	0.14	4.00***
Long-term employment income orientation	0.30	8.34***	0.10	2.6**	0.27	7.88***
Employment anxiety					0.29	8.52***
*R* ^2^	0.20		0.03		0.28	
*F*	31.80***		4.40***		41.59***	
Sex	–0.10	2.70***	–0.05	–1.19	–0.08	−2.43**
Grade	–0.05	–1.49	–0.02	–.41	–0.05	–1.43
School	–0.21	−5.69***	–0.05	–1.23	–0.20	−5.60***
Major	0.13	3.61***	0.12	3.07***	0.10	2.71***
Short-term employment income orientation	–0.22	−5.92***	–0.03	–.65	–0.21	−6.05***
Employment anxiety					0.31	9.04***
*R* ^2^	0.16		0.02		0.26	
*F*	24.02***		3.13***		36.19***	
Sex	–0.07	−1.97**	–0.04	–0.94	–0.06	−1.76*
Grade	–0.05	–1.64	–0.02	–0.41	–0.05	–1.59
School	–0.18	−5.47***	–0.04	–0.91	–0.17	−5.45***
Major	0.13	3.97***	0.12	2.98***	0.10	3.17***
Employment cost avoidance orientation	0.46	13.64***	0.13	3.25***	0.42	13.17***
Employment anxiety					0.27	8.33***
*R* ^2^	0.31		0.04		0.38	
*F*	58.04***		5.20***		65.15***	

*p < 0.1, **p < 0.05, ***p < 0.01.

Second, Model 7, computed using the process macro program, was used to control gender, grade, school type, and major category to test the moderation effect of social support. The results are reported in Table 4, and the interaction between the long-term employment income orientation and social support significantly positively predicts employment anxiety (β = 0.13, *P* < 0.1). The interaction between the short-term employment income orientation and social support significantly positively predicts employment anxiety (β = 0.12, *P* < 0.01). The interaction between the employment cost avoidance orientation and social support negatively predicts employment anxiety (β = –0.04, *P* > 0.1) but not to a significant degree. H3a and H3b are thus verified.

**TABLE 4:** Moderating effect test results.

	Employment anxiety	
	β	*t*
Sex	0.06	–1.43
Grade	–0.05	–0.49
School	–0.03	–0.87
Major	0.10	3.04***
Long-term employment income orientation	0.11	3.17***
Social support	–0.50	−14.78***
Long-term employment income orientation × Social support	0.06	1.77*
*R* ^2^	0.28	
*F*	35.83***	
Sex	–0.05	–1.46
Grade	–0.03	–0.84
School	–0.02	–0.62
Major	0.10	2.89***
Short-term employment income orientation	–0.01	–0.43
Social support	–0.51	−14.90***
Short -term employment income orientation × Social support	0.12	3.29***
*R* ^2^	0.28	
*F*	35.51***	
Sex	–0.04	–1.25
Grade	–0.02	–0.73
School	0.01	–0.36
Major	0.09	2.52**
Employment cost avoidance orientation	0.10	2.99***
Social support	–0.49	−14.56***
Employment cost avoidance orientation × Social support	–0.04	–1.32
*R* ^2^	0.28	
*F*	35.37***	

*p < 0.1, **p < 0.05, ***p < 0.01.

**FIGURE 2:**
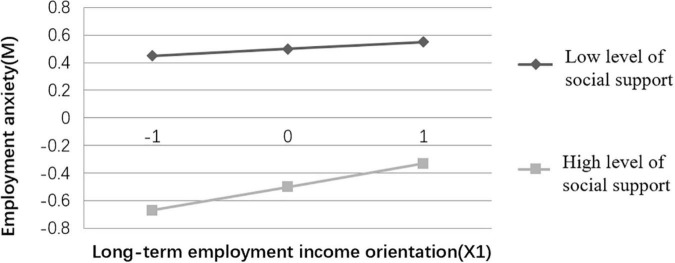
Moderation effects of social support (X1M).

**FIGURE 3:**
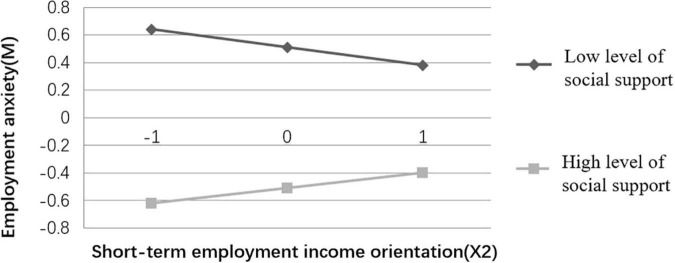
Moderation effects of social support (X2M).

To better explain the moderated mediation model, social support was divided into the high (M + 1 sd) and low groups (M – 1 sd), and simple slope analyses were performed for the two groups, with significant moderation effects (see Figures 2, 3). The results (Figure 2) indicates that for college students with high level of social support, there is a positive and significant correlation between the long-term employment income orientation and employment anxiety, but the prediction is insignificant for college students with low level of social support. Figure 3 reveals that for college students with low levels of social support, the short-term employment income orientation negatively predicts employment anxiety, but for college students with high level of social support, short-term employment income orientation positively predicts employment anxiety, and the both predictions are significant.

Finally, further analysis of the moderation effects of social support reveals that social support also plays a moderated role in mediation effects (see Table 5). With high levels of social support, the mediation effect of employment anxiety between the long-term employment income orientation and choice intention of slow employment is significant; however, this is insignificant at lower levels of social support. The mediation effect of employment anxiety between the short-term employment income orientation and choice intention of slow employment is insignificant under high levels of social support, but it is significant under the low level of social support. The difference of mediation effect between the high and low levels of social support is obvious. The mediation effect of employment anxiety on the employment cost avoidance orientation and choice intention of slow employment is significant at both the high and low levels of social support, although the mediation effect is stronger at higher levels of social support. H4a, H4b, and H4c are thus verified.

**TABLE 5:** Moderated mediation effect.

	Social support	Indirect effect	SE	BootLLCI	BootULCI
X1->M->Y	Low	0.021	0.017	–0.005	0.052
	High	0.074**	0.028	0.029	0.121
X2->M->Y	Low	–0.052**	0.017	–0.090	–0.021
	High	0.040	0.025	–0.009	0.088
X3->M->Y	Low	0.031**	0.016	0.003	0.065
	High	0.058**	0.023	0.018	0.106

X1 indicates long-term employment income orientation. X2 indicates short-term employment income orientation. X3 indicates employment cost avoidance orientation.

*p < 0.1, **p < 0.05, ***p < 0.01.

## Discussion

### Long-term employment income orientation and the choice intention of slow employment

The long-term employment income orientation has a significant positive impact on the choice intention for slow employment, and employment anxiety plays a mediating role, whereas, social support positively moderates the positive impact of long-term employment income orientation on employment anxiety. While pursuing individuality and ideals, some college students focusing on the long-term benefits of employment may plan their careers or improve their employment competitiveness for a period of time after graduation (Jiang and Liu, 2020). Because they do not seek employment in the immediate near term, their willingness to choose slow employment may be higher. Some college students focusing on the long-term benefits of employment may have been confused owing to their lack of confidence in facing graduation and employment. Owing to the increasingly challenging employment situation, it is easy to feel higher employment anxiety, increasing the possibility of choosing slow employment to avoid employment and reduce employment anxiety (Li, 2020; Xia, 2021). Previous studies have found that social support can help reduce employment anxiety (Wu and Lu, 2013; Chen et al., 2020); however, the results of the current study contradict these works. Where there are high levels of social support, a stronger positive correlation can be found between long-term employment income orientation and employment anxiety. This may be because when the long-term income of employment is considered, college students with high level of social support actually experience a certain invisible pressure, which easily produces relatively high employment anxiety. Moreover, only with a high level of social support, owing to more pressure from peers, family, school, and society, college students who are oriented to long-term employment earnings choose slow employment to alleviate anxiety. This shows that social support can’t alleviate employment anxiety, which is different from previous conclusions (Meshi and Ellithorpe, 2021).

### Short-term employment income orientation and the choice intention of slow employment

Because the degree of employment anxiety among college students who pay attention to short-term income as they hunt for job is not only affected by the internal and external environment (Jie, 2007), short-term employment income orientation is insufficient to significantly affect employment anxiety. In addition, it may be that some college students who focus on the short-term income of employment only want to find a job. Owing to the low expectations of employment quality, they focus their attention on the achievement of employment (Liang and Liang, 2012). An increase in the short-term employment income orientation will not cause a significant reduction in employment anxiety. Some previous studies have suggested that social support helps to reduce employment anxiety (Wu and Lu, 2013; Chen et al., 2020), the moderation effect of social support on college students’ short-term employment income orientation and employment anxiety unsupports this conclusion. With low level of social support, college students with a high level of the short-term employment income orientation have lower employment anxiety. However, with a high level of social support, college students with a high level of the short-term employment income orientation experience greater pressure and are prone to a higher level of employment anxiety. Social support plays a significant moderating role between the short-term employment income orientation and Employment anxiety and also in the mediation effect. Under a high level of social support, college students with short-term employment income orientation still have high employment anxiety, which again reveals that social support may not be able to alleviate employment anxiety. Only with a low level of social support, the short-term employment income orientation negatively significantly predicts employment anxiety, and the mediation effect of the short-term employment income orientation on the choice intention of slow employment is significant. When the social support level is low, college students addressed the short-term income of employment are more likely to avoid employment anxiety through their full employment preparation (Liang and Liang, 2012). Owing to the significant mediation effect, college students can also reduce their choice intention of slow employment by reducing their employment anxiety.

### Employment cost avoidance orientation and the choice intention of slow employment

College students who are not oriented to avoiding employment costs are willing to expend more money and spend more time on employment seeking to find a satisfying job, implying that their willingness to find employment is stronger than that of slow employment, and job search time and costs help improve employment opportunities (Pan, 2014); therefore, their choice intention with respect to slow employment is lower. Hence, these college students may be more confident in their own resources, making their employment anxiety relatively low (Jie, 2007). Their low degree of employment anxiety allows these college students to develop a superior mentality and to be more likely to find a job to achieve employment (Li, 2020), and their choice intention of slow employment will be relatively low. The mediation effect of the employment cost avoidance orientation with reference to the choice intention of slow employment demonstrates a significant effect at high and low levels of social support. However, the mediation effect is stronger at high levels of social support, such that college students who focus on employment cost avoidance tend to have relatively high employment anxiety and choose slow employment. This seems inconsistent with the conclusions of the buffering effect and the main effect of social support (Cohen and Wills, 1985; Klineberg et al., 2006), but we believe that this is due to the different employment value characteristics of college students.

## Conclusion

The choice intention of slow employment is related not only to the individual employment of college students but also to the phenomenon of slow employment. This study verified the influence mechanism of employment values on the choice intention of slow employment using a questionnaire survey. First, employment values present college students’ views on employment and have an important impact on the choice intention of slow employment. Second, different employment value orientations have different effects on college students’ choice intention of slow employment. The long-term employment income and employment cost avoidance orientations have significant positive impacts on the choice intention of slow employment, and both can affect the choice intention of slow employment by means of employment anxiety. The short-term employment income orientation has a significant negative impact on the choice intention of slow employment, and the mediation effect for employment anxiety is insignificant. Third, social support plays a crucial moderating role in the impact of employment values on employment anxiety. At high and low levels of social support, the impact of different employment value orientations on the choice intention of slow employment presents different characteristics: at high levels of social support, the long-term employment income orientation has a stronger positive predictive effect on employment anxiety; however, social support changes from low to high, and the predictive effect of the short-term employment income orientation on employment anxiety changes from negative to positive.

### Theoretical implications

This study takes the employment values as the starting point for a study of college students’ choice intention of slow employment, contributing to the relevant research literature on college students’ employment values and slow employment. College students are the key focus group in employment research. The spread of the phenomenon of slow employment among college students is not conducive to achieving fuller and higher-quality employment. However, currently, the researches on slow employment begin from this phenomenon and addressed various reasons and countermeasures of the slow employment phenomenon, and the specific study from the perspective of behavior subject is not perfect. This study supplements that research and these conclusions of can also provide theoretical support for the study of the employment psychology of college students after COVID-19. Based on Hofstede’s long-term and short-term cultural values and Pan’s employment income and cost views (Hofstede, 2011; Pan, 2014), this study segregates employment values into long-term employment income, short-term employment income and employment cost avoidance orientations. We segregated the employment values into three dimensions, expanded the dimensions of employment values, and make the dimension research of employment values more specific. The moderating role of social support in this study is different from the general conclusions of previous social support theories, which enlightens us that the application of social support theory is different in different characteristic groups, which makes a certain exploration for the application scope of social support.

### Practical implications

The impact of employment values on the choice intention of slow employment indicates that if we want to reduce college students’ choice intention of slow employment and promote the realization of fuller and higher-quality employment among them, we should first promote the establishment of good employment values and explain the long-term and short-term benefits of employment. While discussing the establishment of employment values, we should not blindly emphasize the long-term benefits of the spiritual aspects of development space and work–life balance. In an uncertain employment environment, we should help college students think clearly about and understand the long-term benefits of employment, guiding them to realize the long-term benefits of employment, and formulate a series of practical implementation plans. Furthermore, we should help college students correctly understand the short-term employment benefits of material aspects, such as salary, welfare, and working environment and help them establish clearer short-term employment goals to encourage them to actively obtain and achieve full employment.

The mediating role of employment anxiety can enable us to better understand college students’ employment psychology and guide them to seek to actively obtain employment through classification. For college students seeking to understand the long-term benefits of employment, communication should be deployed to help students make career plans more rapidly, improve their career adaptability, reduce their employment anxiety, and shorten the time spent in slow employment (Shu et al., 2021). To better assist students who tend to focus on the expected short-term benefits of employment, we should completely understand the employment needs and challenges that students face, regularly following up on the employment progress of students, and setting up special job search consulting points to provide timely and effective employment guidance and services for college students and help them resolve employment issues.

The fact that social support plays a moderating role shows us that colleges and universities should integrate employment resources with family, enterprises, government, and society to help college students understand the employment situation, emphasize employment dynamics and increase employability. Colleges and universities should develop more exchange activities to promote employment, encourage college students to exchange employment experience and skills, and increase college students’ employment confidence. Colleges and universities can strengthen cooperation with business, enhance the interaction between college students and businesses to lead to employment, broaden the employment channels of college students, and promote the full employment of college students. Families and colleges and universities can build communication platforms among themselves to provide appropriate and timely help for the different needs of college students and solve the spiritual and material problems in employment. Furthermore, colleges and universities, families, news media, and other agents should jointly create a positive employment atmosphere, providing appropriate encouragement and support while reducing pressure on college student, their employment anxiety, and the ability of slow employment owing to anxiety to breed. Moreover, we should mobilize the employment enthusiasm of college students and enable them to eliminate slow employment or shorten its time.

### Research limitations and future directions

This study has several limitations as well. First, family conditions have a crucial impact on college students’ choice of slow employment. Although material support is reflected in social support, it has never been examined separately or controlled. More factors must be considered in subsequent research. Second, there is still room for improvement in the measurement of employment values with reference to the research of Hofstede (2011) and Pan (2014). Third, the choice intention of slow employment is measured according to its definition. It only measures the willingness of several behaviors contained in slow employment, and there may be potential slow employment willingness that has not been measured. Fourth, this is a cross-sectional study, and its prediction accuracy of college students’ subsequent slow employment behavior is not high. Finally, this study only examines the role of employment anxiety as a mediating variable to explore mediation mechanisms, but there may be other mechanisms as well.

The results and limitations of this study indicate some promising directions for future research. In particular, it would be interesting to study the moderating role of different social subjects (parents, siblings, friends, classmates, teachers, and others) to deeply investigate the influence degree of different social subjects. Another direction would be to find the different influence mechanisms to analyze the impact of employment values on the choice intention of slow employment. If we investigate college graduates who exhibit the behavior of slow employment, we can also explore the influence of employment values on the behavior of slow employment and its influence mechanism. Briefly, influenced by the external environment, college students’ slow employment is a group phenomenon in which many issues deserve our attention and examination.

## Data availability statement

The raw data supporting the conclusions of this article will be made available by the authors, without undue reservation.

## Author contributions

TW contributed to conceptualization, data analysis, writing, and revision. SL contributed to data collection, theoretical model construction, and original draft preparation. Both authors contributed to the article and approved the submitted version.

## Footnotes

1 Zhaopin Ltd., a professional online recruitment service company, conducts an annual employment survey of college students in China. In the 2022 survey, a survey report on the employment of college students was issued based on 500,000 questionnaires returned by college students nationwide. https://www.sgpjbg.com/baogao/74831.html.
